# Alpha-lipoic acid prevents atrial electrical and structural remodeling via inhibition of NADPH oxidase in a rabbit rapid atrial pacing model

**DOI:** 10.55730/1300-0144.5445

**Published:** 2022-03-19

**Authors:** Lei CHEN, Wen GAO, Yameng SHAO, Chenggang LI, Yuan LU

**Affiliations:** 1 Department of Cardiology, the Affiliated Hospital of Xuzhou Medical University, Xuzhou, China; 2 Department of Cardiology, Xuzhou Municipal Hospital Affiliated to Xuzhou Medical University, Xuzhou, China; 3 Department of Cardiology, Xuzhou New Health Geriatric Hospital, Xuzhou, China

**Keywords:** NADPH oxidase, rapid atrial pacing, reactive oxygen species, oxidative stress, alpha-lipoic acid

## Abstract

**Background/aim:**

Alpha-lipoic acid (ALA) is a natural compound, one of the natural antioxidants with high activity. In the NADPH oxidase family, NADPH oxidase 4 (NOX4) is an important subunit participating in the production of ROS. NADPH oxidase 2 (NOX2) can form active NADPH oxidase complexes when binding to several other subunits in the cytoplasm, and NOX2 is its major functional subunit. Rapid atrial pacing (RAP) model was constructed to study the effects of ALA on electrical and structural remodeling in rabbits.

**Materials and methods:**

Thirty rabbits were divided into SHAM group, RAP group and ALA+RAP group. Their right atriums were paced at a speed of 600 beats/min for 12 h in the RAP and ALA+RAP groups, and the atrial effective refractory period (AERP) and AERP frequency adaptability were determined during the pace. In ALA+RAP group, ALA (30 mg/kg) was administered intraperitoneally daily to the rabbits for 3 days before RAP. Atrial tissue was collected from each group to detect malondialdehyde (MDA), superoxide dismutase (SOD) and reactive oxygen species (ROS) to observe the effect of oxidative stress. The pathological structure of the atrial tissue was observed through hematoxylin-eosin (HE) staining. Ultrastructural changes in the atrial myocytes were observed by transmission electron microscopy (TEM), and the expression levels of Nox2 and Nox4 were detected by immunohistochemistry, western blot and ELISA.

**Results:**

AERP gradually shortened, while ALA injection could remarkably delay this process. HE staining showed that the most of the nuclei appeared normal, the myocardial fibers did not show ruptures, and their arrangement was slightly ordered, and myofilament dissolution and mitochondrial swelling and deformation were rarely observed by TEM in the ALA+RAP group. Compared with the RAP group, the contents of MDA and ROS were reduced, SOD activity was enhanced, and the expression of NOX2 and NOX4 was decreased in the ALA+RAP group.

**Conclusion:**

ALA can inhibit atrial electrical remodeling and structural remodeling, and its mechanism may be related to inhibiting the activity of NADPH oxidase.

## 1. Introduction

Atrial fibrillation (AF) is one of the most common arrhythmias encountered in the clinic [1]. Atrial fibrillation-induced symptoms and complications can severely affect the quality of life and cause mortality [2–4]. Long-term maintenance of the sinus rhythm of AF remains a considerable challenge due to its complicated pathogenesis. The available methods for controlling the AF rhythm include antiarrhythmic drugs and catheter ablation, both of which have obvious limitations [5, 6]. Upstream treatment of AF is a novel method introduced in recent years and it aims to achieve primary and secondary prevention of AF [7]. It was first established in 2010 by the European Society of Cardiology to be a new method and strategy to prevent AF [8]. However, the precise mechanism of the genesis and development of AF remains incompletely known. Therefore, additional research on the genesis and maintenance of AF is of crucial importance.

Many studies have indicated that atrial remodeling is the central link for the genesis and maintenance of AF, which mainly includes atrial electrical remodeling, structural remodeling and contractile remodeling [9–11]. As research continues in recent years, growing evidence has indicated that oxidative stress plays an important role in atrial remodeling [12–14]. In oxidative stress, superoxide dismutase (SOD) is the most important enzyme in the first-line cellular defense [15], and it is extensively distributed in all tissue and cells. Malondialdehyde (MDA) is the end byproduct of lipid peroxidation in oxidative stress [16], which is frequently treated as a detection index of lipid peroxidation degree. In addition, a large amount of reactive oxygen species (ROS) will be produced in the case of oxidative stress, which plays a vital role in AF [17,18]. Consequently, effective regulation of ROS levels in vivo has become an important intervention target in the upstream treatment of AF. ROS has numerous sources in vivo, but many studies have suggested that nicotinamide adenine dinucleotide phosphate (NADPH) oxidase is its primary source [19–21]. In the NADPH oxidase family, NOX4 is an important subunit participating in the production of ROS [22, 23]. Gp91phox (NOX2) and p22phox subunits are located on the plasma membrane, which can form active NADPH oxidase complexes when binding to several other subunits (including NOX4) in the cytoplasm, and NOX2 is its major functional subunit. Plenty of evidence from clinical and experimental research has indicated that ROS plays a core role in numerous cardiovascular diseases (CVD’s, including AF). However, the clinical effects of antioxidant stress treatment are not satisfactory. This may be because a general ROS scavenger can only neutralize one or several forms of ROS, while other ROS forms still exert a proarrhythmic effect [24,25]. Therefore, a more comprehensive ROS scavenger or a targeted therapy specific to each ROS source is required.

Alpha-lipoic acid (ALA) is a natural compound, one of the natural antioxidants with high activity [26]. Compared with other natural antioxidants such as vitamin C and carotene, ALA is water and lipid soluble, and it can exist in the cytoplasm and cell membrane. Moreover, it has been applied in diabetes [27], CVD [28] and liver-kidney disease [29]. ALA can eliminate ROS and reduce the production of ROS by targeting NADPH oxidase [30–32]. Therefore, compared with general antioxidants, ALA cannot only more comprehensively eliminate active substances in vivo but also target NADPH oxidase. So far, research on the role of ALA in CVD has been reported, but only a limited number of studies have reported on its effects on arrhythmia. This study constructed a rapid atrial pacing (RAP) rabbit model to evaluate the effect of ALA on atrial remodeling in AF and its possible mechanism. Hopefully, this research can provide a new possibility for the clinical treatment of AF.

## 2. Materials and methods

### 2.1. Animals

In this study, 30 healthy New Zealand white rabbits weighing 2.9 ± 0.2 kg (2.7–3.1 kg) were selected regardless of sex. All rabbits were randomly assigned to the SHAM group (n = 10), the RAP group (n = 10), or the ALA + RAP group (n = 10). Rabbits were housed under conventional conditions and exposed to light-dark cycle of 12 h with free access to water and food. Continuous care was provided throughout the study to ensure prompt intervention when needed. All rabbits were obtained from Xuzhou Medical University Animal Center. This study was approved by the Animal Ethics Committee of Xuzhou Medical University Animal Center.

### 2.2. ALA solution preparation

ALA was purchased from Yuanye Biotechnology (Shanghai, China). ALA (80 mg/mL) was dissolved in 10% ethanol and sterilized by filtering. ALA solutions were prepared immediately before use. In the ALA+RAP group, dissolved ALA (0.375 mL/kg) was administered intraperitoneally daily (i.p.) to the rabbits for 3 days before RAP [31]. In the remaining groups, equal volume of normal saline was injected intraperitoneally.

### 2.3. Construction of the rapid atrial pacing (RAP) model

First, 1% pentobarbital sodium solvent was injected slowly through the ear vein at 2 mL/kg. After successful anesthesia induction, the anesthesia was maintained through an indwelling needle in the ear vein and a constant speed micropump. An incision was made in the middle of the neck, the trachea was isolated, tracheal intubation was conducted, and an animal ventilator was connected to assist in ventilation. The right internal jugular vein was isolated, and distal shaping of the 10-pole coronary sinus electrode was conducted, which was sent to the right atrium through the 6F sheathing canal, and the chest lead V1 was connected to the distal end of the coronary sinus buttock line.

The BL-420s biological function experimental system (TECHMAN, Chengdu, China) was connected, and continuous stimuli were released at a frequency 10%–20% higher than the sinus rhythm. Microadjustment of the coronary sinus electrode direction was performed so that the stimulating electrode adhered to the right atrium. Then, 1:1 atrial and ventricular conduction observed simultaneously in the intracavity lead and the body surface lead electrocardiograms revealed complete atrial pacing. The position of the coronary sinus electrode was maintained, and 12 h continuous stimuli were carried out by pacing at a rate of 600 times/min at a pressure twice that of the pacing threshold. After pacing, the rabbits were euthanized by intravenous injection of 100 mg/kg pentobarbital sodium.

### 2.4. AERP determination

The 2-fold diastolic pacing threshold was treated as the output voltage, and the pulse width was set at 0.5 ms to measure AERP under the basic condition. Programmed premature extrastimulation (S1S2) was adopted at a stimulation frequency of 8:1. Decreasing scanning was carried out at S1S2 interphase at a step length of 10 ms and a scanning interval of 30 s. The refractory period was defined as the longest S1S2 interphase that S2 did not induce atrial activation in the time of S1S2 stimulation. AERP was measured 3 times, and the mean was treated as the baseline AERP value. AERP200 and AERP150 at the S1S1 of 200 ms and 150 ms were measured, respectively. The frequency adaptability is expressed as AERP200 − AERPl50. Rapid atrial pacing was performed at the rate of 600 bpm in RAP group and ALA+RAP group. The AERP was measured repeatedly at 0h, 4h, 8h and 12h after pacing. In SHAM group, AERP was only measured at the same time, but not rapid pacing.

### 2.5. Tissue processing

The hearts were excised and washed immediately with buffered saline (pH 7.4) to remove any red blood cells and clots. The tissues were then weighed and homogenized in normal saline (0.9%) for 2 min. The heart tissue was first divided into three parts, one of which was homogenized to complete the determination of WB, ELISA and oxidative stress indexes. All homogenate samples were centrifuged for 20 min at 3000 rpm at 4 °C in a refrigerated centrifuge to remove debris. The other part of the tissue was fixed and sliced to complete H&E staining and immunohistochemistry. The last part was fixed and sent to Neuroelectrometer Experimental Center of Xuzhou Medical University for processing to complete transmission electron microscopy observation.

### 2.6. Detection of NOX4 and oxidative stress-related parameters

Atrial tissue was collected from each group to detect malondialdehyde (MDA), superoxide dismutase (SOD) and reactive oxygen species (ROS) to observe the effect of oxidative stress. After the tissue homogenate was completed, the levels of MDA, SOD and ROS were detected by using the respective assay kits according to the instructions. ROS detection kits were purchased from Beyotime Biotechnology (Shanghai, China). MDA detection kits and SOD activity assay kits were obtained from Nanjing Jiancheng Bioengineering Institute (Nanjing, China). The levels of the NOX4 protein were determined by ELISA. The ELISA kits for the protein Nox4 were purchased from Westang Biotechnology (Shanghai, China).

### 2.7. Histopathological analysis

Samples of heart tissues were washed immediately with saline (0.9%) and fixed in 10% phosphate buffered formalin solution for 24 h. The tissues were then dehydrated in ascending grades of ethyl alcohol, cleared with xylene, and embedded in paraffin. Paraffin blocks were cut by a microtone to prepare 5 μm thick sections. Sections were stained with hematoxylin-eosin (H&E) to stain the nucleus and cytoplasm.

### 2.8. Ultrastructural changes

After precooling the relevant instruments at 4 °C, the samples of heart tissues were washed with saline (0.9%) then cut into 1 × 1 × 1 mm^3^ pieces and placed immediately in 4% glutaraldehyde at 4 °C for 4 h. Afterward, the samples were sent to the Neuroelectrometer Experimental Center of Xuzhou Medical University, sliced and stained, and observed by transmission electron microscopy (HITACHI, Japan).

### 2.9. Immunohistochemistry

For the detection of NOX-2, paraffin sections were deparaffinized, rehydrated and washed in 0.05 M Tris-buffered saline (TBS) pH 7.6. Immunostaining was performed using a rabbit polyclonal antibody (anti-NOX2) at a dilution of 1:250 for 1 h at room temperature. Negative controls were performed by omission of the primary antibody. Then, the sections were incubated with NovoLink Polymer Detection System (product No. RE 7280-K; Leica Biosystems, Newcastle, UK) for 30 min to detect any tissue-bound primary antibody. Sections were further incubated with the substrate/chromogen, 3,3-diaminobenzidine (DAB), prepared from Novocastra DAB chromogen (50 μl) and 1 mL of NovoLink Substrate Buffer (Polymer). Washing with TBS (0.05 M, pH 7.6) twice for 5 min was performed following each step of incubation. Immunostained sections were then counterstained with Mayer’s hematoxylin. Finally, sections were dehydrated rapidly in ascending grades of ethanol, cleared in xylene, and mounted in DPX mounting medium. The stained sections were evaluated and photographed using an Olympus BX51 bright field microscope equipped with an Olympus DP72 camera (Olympus Corporation, Japan). Anti-NOX2 was purchased from Bioss Biotechnology (Beijing, China).

### 2.10. Western blot

The whole-cell lysates of the cortical samples (N = 3 for each group) were obtained using tissue protein extraction kits supplemented with protease inhibitors (Roche, IN, USA). The total protein concentration was determined using BCA kits. An equal amount of protein was separated by electrophoresis in 10% sodium dodecyl sulfate polyacrylamide gels and then transferred to nitrocellulose membranes. After washing with TBST, the blots were then incubated with the respective secondary antibodies for 2 h and developed by chemiluminescence. Relative optical densities of the blots were quantified by densitometry. Rabbit polyclonal anti-NOX4 antibody was obtained from Novus Biologicals (Littleton, USA).

### 2.11. Statistical analysis

SPSS 19.0 statistical software (SPSS, Chicago, Illinois, USA) was adopted for statistical analysis. Measurement data were expressed as mean ± standard deviation. Data among multiple groups at all time points were compared using one-way analysis of variance. Differences in continuous repeated measuring data were compared by the repeated data analysis of variance. Further pairwise comparison was carried out using a Dunnett t-test. Differences were considered significant when p <0.05.

## 3. Results

### 3.1. Electrical remodeling-related indicators

#### 3.1.1. Effect of ALA on AERP

Differences in AERP200ms and AERP150ms among the three groups under the basic condition (before pacing) were not significant (p > 0.05). AERP200ms and AERP150ms had no obvious variation trend with an extension in time within 12 h in the SHAM group, while they were gradually shortened with the extension of pacing time within 12 h in both the RAP group and the ALA+RAP group (p < 0.05). Compared with the SHAM group, AERP200ms and AERP150ms were significantly shortened after rapid pacing in the RAP group (p < 0.05). Compared with the RAP group, AERP200ms and AERP150ms in the ALA+RAP group were increased at the corresponding time points (p < 0.05) (Tables 1 and 2).

#### 3.1.2. Effect of ALA on AERP frequency adaptability

AERP frequency adaptability in the SHAM group indicated no distinct changes with an extension of pacing time within 12 h, while they were notably changed in the RAP group and the ALA+RAP group (p < 0.05). Compared with the SHAM group, AERP frequency adaptability in the RAP group was shortened at corresponding time points (p < 0.05). Compared with the RAP group, AERP frequency adaptability in the ALA+RAP group was increased at corresponding time points (p < 0.05). AERP frequency adaptability was shortened in the ALA+RAP group at corresponding time points compared with the SHAM group (p < 0.05) (Table 3).

### 3.2. Structural remodeling-related indicators

#### 3.2.1. HE staining

**Figure 1:**
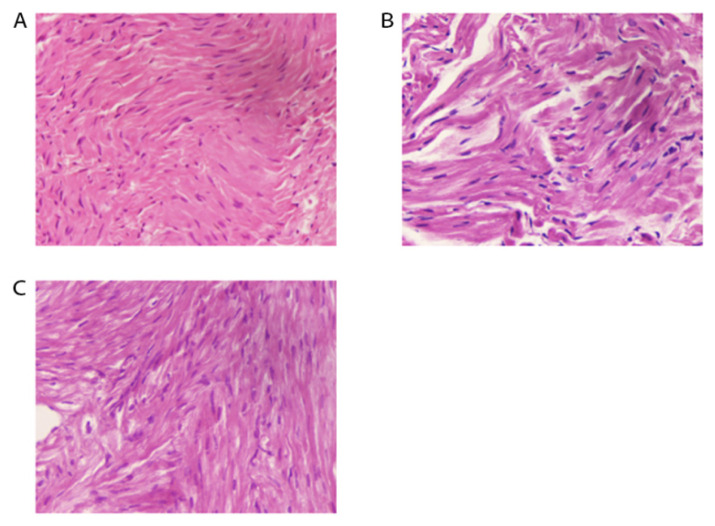
Light photomicrographs of the rabbit’s heart stained with hematoxylin and eosin (×200); (A) Myocardial fibers and nuclei in the SHAM group; (B) Myocardial fibers and nuclei in the RAP group; (C) Myocardial fibers and nuclei in the ALA+RAP group.

The rabbits in the SHAM group (Figure 1a) showed orderly myocardial fibers and normal nuclei. Atrial muscle cell hypertrophy could be seen in the RAP group (Figure 1b) along with irregularly sized nuclei, many nuclei showed pyknosis, there were disorderly myocardial fibers, and myocardial fibers rupturing and dissolving in some regions. Connective tissue accumulated between the muscle fibers, and there were gaps between myocardial cells. Pathological changes in the ALA+RAP group (Figure 1c) were milder than those in the RAP group. Most of the nuclei appeared normal, the myocardial fibers did not show ruptures, and their arrangement was slightly ordered.

#### 3.2.2. Effect of ALA on ultrastructural changes in each group

**Figure 2:**
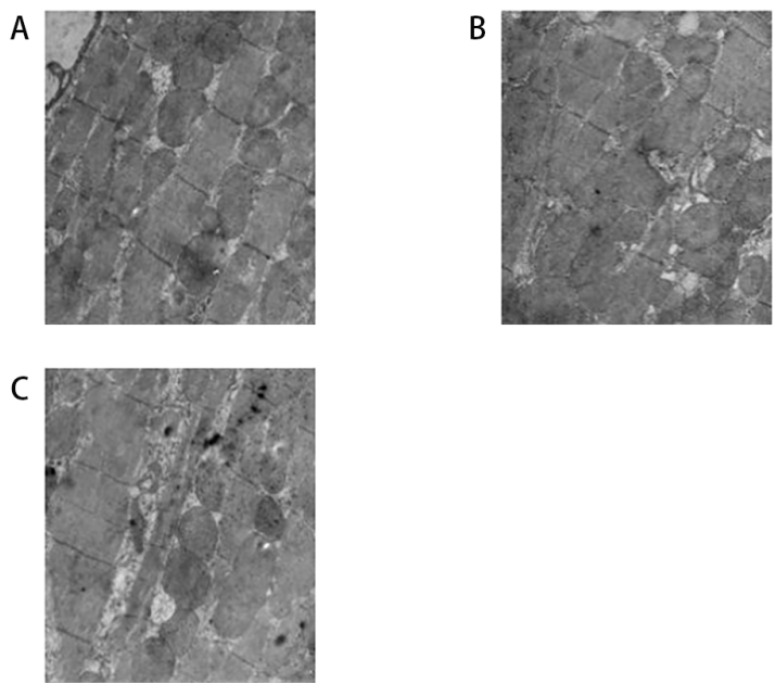
Ultrastructural changes of the heart with transmission electron microscopy; (A) The ultrastructure in the SHAM group; (B) The ultrastructural changes in the RAP group; (C) The ultrastructural changes in the ALA+RAP group.

As shown in Figure 2, the tissue sections from the SHAM group (Figure 2a) showed a normal ultrastructure, while it was severely injured in the RAP group (Figure 2b), characterized by myofilament dissolution and mitochondrial swelling and deformation. While the myofilament dissolution and mitochondrial swelling and deformation were rarely observed in the ALA+RAP group (Figure 2c) compared with the RAP group (Figure 2b).

### 3.3. Effect of ALA on oxidative stress in each group

As shown in Table 4, compared with the SHAM group, the contents of MDA and ROS were significantly increased in the RAP group (p < 0.05), while SOD activity was decreased (p < 0.05). Compared with the RAP group, the contents of MDA and ROS were reduced in the ALA+RAP group (p < 0.05), while SOD activity was enhanced (p < 0.05).

### 3.4. Effects of ALA on NOX2 and NOX4 in each group

#### 3.4.1. Western blot

**Figure 3:**
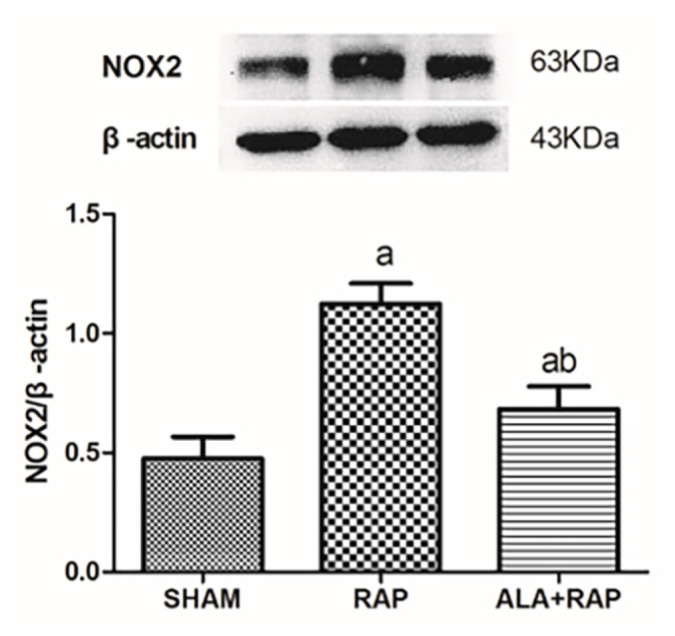
a, *p*< 0.05 compared with the SHAM group; b, *p*< 0.05 compared with the RAP group.

**Figure 4:**
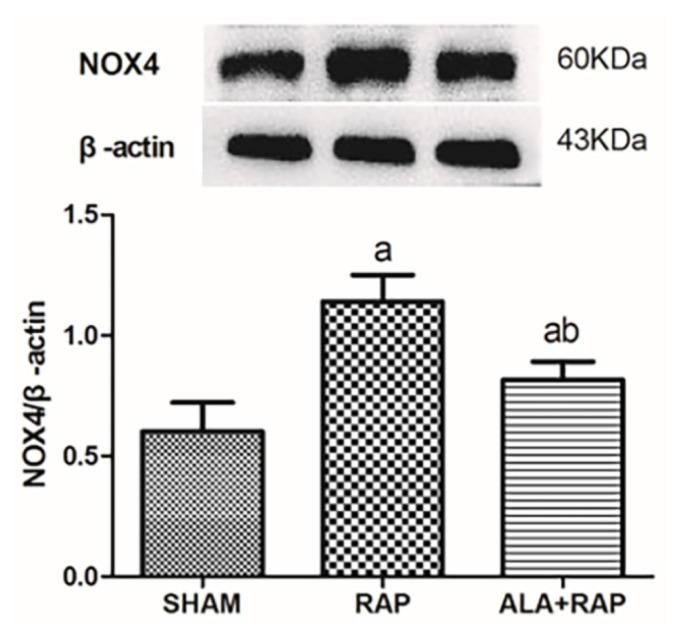
a, *p* < 0.05 compared with the SHAM group; b, *p* < 0.05 compared with the RAP group.

Compared with the SHAM group, expression of NOX2 and NOX4 in the atrial muscle tissue in the RAP group was upregulated (p < 0.05). Thus, RAP induced the upregulation of NOX2 and NOX4. Compared with the RAP group, expression of NOX2 and NOX4 in the atrial tissue in the ALA+RAP group was decreased (p < 0.05) compared with the SHAM group (Tables 5 and 6, Figure 3 and 4).

#### 3.4.2. Immunolocalization of NOX2

**Figure 5:**
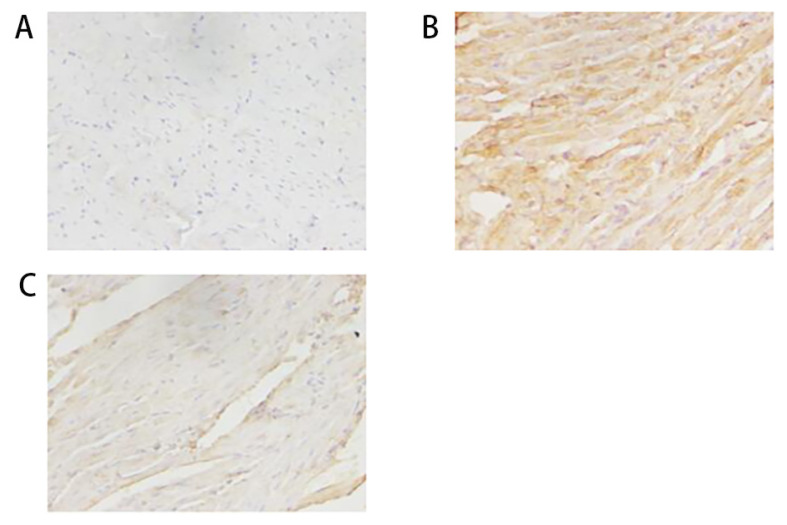
Immunolocalization of NOX2 in myocardial tissue (×400); (A) Immunolocalization of NOX2 in the SHAM group; (B) Immunolocalization of NOX2 in the RAP group; (C) Immunolocalization of NOX2 in the ALA+RAP group.

As shown in Figure 5, the yellow staining indicates positive NOX2 expression. Compared with the SHAM group (Figure 5a), the RAP group (Figure 5b) had a markedly enlarged stained area, along with notably deepened yellow staining. Compared with the RAP group (Figure 5b), the staining area in the ALA+RAP group (Figure 5c) was distinctly reduced, and the yellow staining was notably lightened.

### 3.5. ELISA

**Figure 6:**
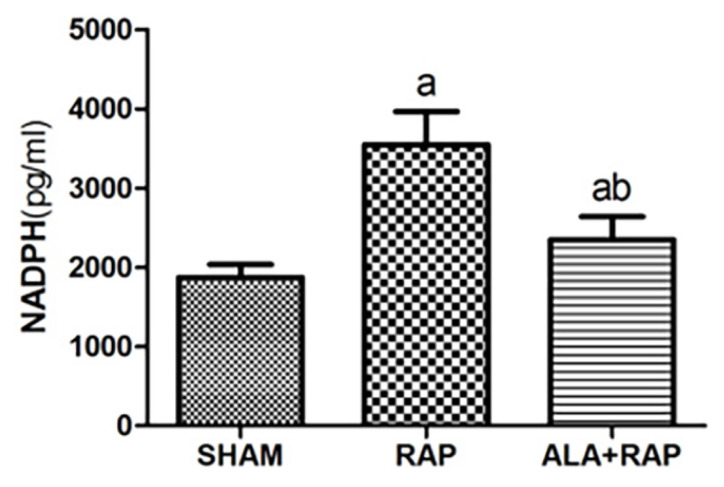
a, *p* < 0.05 compared with the SHAM group; b, *p* < 0.05 compared with the RAP group.

Compared with the SHAM group, the NADPH oxidase (NOX4) expression level in the RAP group was notably upregulated (p < 0.05), and compared with the RAP group, the expression of NADPH oxidase (NOX4) in the ALA+RAP group was significantly downregulated (p < 0.05). Compared with the SHAM group, the expression of NADPH oxidase (NOX4) in the ALA+RAP group was slightly upregulated (p < 0.05) (Table 7, Figure 6).

## 4. Discussion

In this study, the frequency adaptability of AERP and AERP were measured to evaluate atrial electrical remodeling [34]. After RAP stimulation, electrical remodeling-related indicators in the RAP group developed remarkable changes in the early stage, which manifests as a shortened AERP and a loss of frequency adaptability. The results of HE staining and the SEM showed that compared with the SHAM group, rabbit atrial ultrastructure and pathological structures were notably changed in the RAP group. This further verifies that electrical remodeling and structural remodeling are closely related to the genesis and development of AF.

Oxidative stress participates in atrial electrical remodeling and structural remodeling, thus giving rise to lesions like atrial fibrosis [35,36]. This experiment constructed an RAP rabbit model, and atrial muscle oxidative stress-related indicators, namely, ROS, SOD and MDA, were detected to reflect oxidative stress in the atrial muscle tissue at the time of AF. Among them, ROS is one of the major causes of oxidative stress, which plays a vital role in the genesis and maintenance of AF [37]. As was found in this experiment, among the oxidative stress-related indicators, the ROS and MDA content increased while SOD activity decreased in the RAP group compared with the control group. Thus, RAP can reduce SOD activity, destroying the first-line defense of ROS and interfering with the balance between oxidation and antioxidation regulation, which then increases ROS and MDA content. These findings indicated that RAP could induce oxidative stress injury, which has further verified that oxidative stress is closely related to AF.

In addition, many studies have reported that antioxidants can effectively improve atrial electrical remodeling and structural remodeling [38,39]. However, their clinical effects remain unsatisfying. Therefore, we are looking forward to searching for a better upstream therapeutic agent or treatment for AF. NADPH oxidase is a major source of ROS in the body, the enhanced activity of which is closely associated with the genesis and development of AF [40–42]. In this experiment, compared with the SHAM group, the expression levels of nox2 and nox4 in the atrial muscle of the RAP group were distinctly upregulated. This suggests that RAP can enhance NADPH oxidase activity, thus promoting ROS production. Consequently, NADPH oxidase targeted therapy may be a novel effective treatment for preventing AF.

ALA is a naturally existing thiol antioxidant that can eliminate most of the active substances produced in the body as compared with general antioxidants. Meanwhile, it can inhibit NADPH oxidase. Research on the role of ALA in CVD has increased gradually due to its unique features. It was discovered that ALA exerts antioxidative effects in CVDs through multiple pathways, thus alleviating the genesis and progression of disease [43–45]. However, research on its role in arrhythmia, especially AF, is rarely reported. In this study, we observed oxidative stress-related indicators and discovered that the ALA+RAP group notably reduced the levels of oxidative stress induced by RAP. Through HE staining and the SEM results, compared with the RAP group, the pathological structures in the ALA+RAP group were significantly improved. Comparisons of the AERP and AERP frequency adaptability among all groups showed that ALA could delay RAP-induced changes in AERP and AERP frequency adaptability. Furthermore, the results of immunohistochemistry, Western blot and ELISA showed that compared with the RAP group, the ALA+RAP group had reduced expression of the NOX2 and NOX4 proteins. Consequently, we speculate that ALA can improve atrial electrical remodeling and structural remodeling in an RAP model, thus effectively preventing the genesis and progression of AF. Apart from antioxidative effects such as the direct inactivation of ROS, its mechanism of action may be to partially inhibit NADPH oxidase activity and reduce ROS production, thus improving atrial remodeling in this RAP model. It was verified in this experiment that ALA can directly inactivate ROS in an RAP rabbit model. In addition, it can partly inhibit NADPH oxidase activity and reduce ROS production, thus delaying the progression of AF. These results have provided a certain theoretical foundation for the antioxidant treatment of AF.

There are some limitation in this study. First of all, because this is an invasive and open atrial rapid pacing model, it may be difficult to maintain a longer period of time during rapid atrial pacing, but this invasive pacing mode also ensures that the atrium can be paced more effectively. Second, our research content can continue to expand, such as inflammatory response, so as to clarify the specific mechanism of ALA and NADPH in atrial fibrillation, which will continue to be carried out in our future research. Finally, if this study has a larger sample size and more experimental groups, then it may have more promotion and application value.

In conclusion; this article demonstrates the electrical and structural remodeling of the atrium in a rapid atrial pacing model. This study used lipoic acid for the first time in atrial rapid pacing models and proved that ALA intervention could inhibit atrial electrical remodeling and structural remodeling. Its mechanism may be to reduce oxidative stress injury by inhibiting the activity of NADPH oxidase.

## Figures and Tables

**Table 1:** Effect of alpha-lipoic acid on AERP200ms (n=10, χ̄±s, ms).

Group	0h	4h	8h	12h
SHAM	115.60 ± 6.14	115.20 ± 6.09	115.95 ± 5.19	114.45 ± 7.58
RAP	116.84 ± 3.78	99.84 ± 5.05^a^^b^	87.92 ± 8.42^a^^b^	79.49 ± 7.83^a^^b^
ALA+RAP	116.92 ± 3.94	104.76 ± 9.96^a^^b^^c^	100.77 ± 9.85^a^^b^^c^	96.90 ± 4.43^a^^b^^c^

ms, millisecond; h, hour;

a , p< 0.05 compared with the 0h of this group;

b , p< 0.05 compared with the SHAM group;

c , p< 0.05 compared with the RAP group.

**Table 2:** Effect of alpha-lipoic acid on AERP150ms (n=10, χ̄±s, ms).

Group	0h	4h	8h	12h
SHAM	104.24 ± 6.63	103.62 ± 6.06	104.63 ± 5.26	103.97 ± 6.41
RAP	105.83 ± 4.05	89.43 ± 5.00^a^^b^	80.29 ± 8.36^a^^b^	76.07 ± 7.88^a^^b^
ALA+RAP	105.57 ± 3.87	94.28 ± 10.13^a^^b^^c^	91.87 ± 10.00^a^^b^^c^	89.39 ± 4.78^a^^b^^c^

ms, millisecond; h, hour;

a , p< 0.05 compared with the 0h of this group;

b , p< 0.05 compared with the SHAM group;

c , p< 0.05 compared with the RAP group.

**Table 3:** Effect of alpha-lipoic acid on AERP frequency adaptability (n=10, χ̄±s, ms).

Group	0h	4h	8h	12h
SHAM	11.36 ± 0.90	11.58 ± 0.25	11.32 ± 0.28	11.49 ± 0.35
RAP	11.01 ± 2.29	10.41 ± 0.19^b^	7.62 ± 0.22^a^^b^	3.42 ± 0.36^a^^b^
ALA+RAP	11.35 ± 0.80	11.48 ± 0.30^c^	8.90 ± 0.25^a^^b^^c^	7.26 ± 0.63^a^^b^^c^

h, hour;

a , p< 0.05 compared with the 0h of this group;

b , p< 0.05 compared with the SHAM group;

c , p< 0.05 compared with the RAP group.

**Table 4:** Effects of ALA on MDA, SOD and ROS (n=10, χ̄±s).

Group	MDA (nmol/mgprot)(mmol/mgprot)	SOD (U/ mgprot)	ROS (μmol/mg)
SHAM	1.30 ± 0.33	432.19 ± 2.65	0.004 ± 0.001
RAP	4.52 ± 0.28 a	107.50 ± 3.45 a	0.016 ± 0.002 a
ALA+RAP	2.44 ± 0.23^a^^b^	302.09 ± 6.14^a^^b^	0.009 ± 0.001^a^^b^

a , p< 0.05 compared with the SHAM group;

b , p< 0.05 compared with the RAP group.

**Table 5:** Effect of ALA on NOX2.

Group	NOX2/β-actin
SHAM	0.47 ± 0.09
RAP	1.12 ± 0.09^a^
ALA+RAP	0.68 ± 0.10^a^^b^

a , p< 0.05 compared with the SHAM group;

b , p< 0.05 compared with the RAP group.

**Table 6:** Effect of ALA on NOX4.

Group	NOX4/β-actin
SHAM	0.60 ± 0.12
RAP	1.14 ± 0.11^a^
ALA+RAP	0.82 ± 0.07^a^^b^

a , *p* < 0.05 compared with the SHAM group;

b , *p* < 0.05 compared with the RAP group.

**Table 7:** Effect of ALA on NADPH oxidase (NOX4).

Group	NADPH oxidase (pg/mL, n=10)
SHAM	1872.46 ± 161.73
RAP	3549.90 ± 413.94^a^
ALA+RAP	2348.67 ± 289.32^a^^b^

a , *p* < 0.05 compared with the SHAM group;

b , *p* < 0.05 compared with the RAP group.
